# Genome-Wide Association Study Identifies Genomic Loci Affecting Filet Firmness and Protein Content in Rainbow Trout

**DOI:** 10.3389/fgene.2019.00386

**Published:** 2019-05-03

**Authors:** Ali Ali, Rafet Al-Tobasei, Daniela Lourenco, Tim Leeds, Brett Kenney, Mohamed Salem

**Affiliations:** ^1^Department of Biology and Molecular Biosciences Program, Middle Tennessee State University, Murfreesboro, TN, United States; ^2^Computational Science Program, Middle Tennessee State University, Murfreesboro, TN, United States; ^3^Department of Biostatistics, University of Alabama at Birmingham, Birmingham, AL, United States; ^4^Department of Animal and Dairy Science, University of Georgia, Athens, GA, United States; ^5^National Center for Cool and Cold Water Aquaculture, Agricultural Research Service, United States Department of Agriculture, Kearneysville, WV, United States; ^6^Division of Animal and Nutritional Sciences, West Virginia University, Morgantown, WV, United States

**Keywords:** trout, muscle, firmness, softness, protein, GWAS, WssGBLUP, QTL

## Abstract

Filet quality traits determine consumer satisfaction and affect profitability of the aquaculture industry. Soft flesh is a criterion for fish filet downgrades, resulting in loss of value. Filet firmness is influenced by many factors, including rate of protein turnover. A 50K transcribed gene SNP chip was used to genotype 789 rainbow trout, from two consecutive generations, produced in the USDA/NCCCWA selective breeding program. Weighted single-step GBLUP (WssGBLUP) was used to perform genome-wide association (GWA) analyses to identify quantitative trait loci affecting filet firmness and protein content. Applying genomic sliding windows of 50 adjacent SNPs, 212 and 225 SNPs were associated with genetic variation in filet shear force and protein content, respectively. Four common SNPs in the ryanodine receptor 3 gene (RYR3) affected the aforementioned filet traits; this association suggests common mechanisms underlying filet shear force and protein content. Genes harboring SNPs were mostly involved in calcium homeostasis, proteolytic activities, transcriptional regulation, chromatin remodeling, and apoptotic processes. RYR3 harbored the highest number of SNPs (*n* = 32) affecting genetic variation in shear force (2.29%) and protein content (4.97%). Additionally, based on single-marker analysis, a SNP in RYR3 ranked at the top of all SNPs associated with variation in shear force. Our data suggest a role for RYR3 in muscle firmness that may be considered for genomic- and marker-assisted selection in breeding programs of rainbow trout.

## Introduction

Aquaculture continues to experience rapid growth worldwide. However, for a sustainable industry, there is a need to produce fish filets with consistent quality and high value. Consumer attitude toward fish is influenced by nutritional and sensory attributes, including filet firmness (Bonneau and Lebret, 2010). Firmness is one of the most important quality attributes that determines consumer satisfaction toward the product; and, it is affected by many intrinsic and extrinsic factors (Destefanis et al., 2008). These factors include prerigor muscle processing, production and storage temperature, chilling protocols, genotype, handling stress, collagen content, extent of proteolysis, and the proximate composition of muscle (Castañeda et al., 2005; Bahuaud et al., 2010; Grześ et al., 2017). Filet softness shares common causes but should not be confused with gaping that results from tearing the connective tissue between muscle layers and weakening of the interface between the myotome and the myosepta causing slits in the filet (Jacobsen et al., 2017). Previous studies in farmed European whitefish showed that filet firmness is a heritable trait (0.30 ± 0.09); whereas, gaping seems to be not heritable (Kause et al., 2011). Gaping is affected by a range of perimortem harvest and handling factors and postmortem handling practices. In other words, there is a great opportunity for uncontrolled, random variation that makes elucidation of the genetic control of gaping a challenge. Loss of filet firmness and gaping contribute to downgrading during the secondary processing of filet causing economic loss for the industry (Torgersen et al., 2014; Jacobsen et al., 2017). The increased level of stress has been reported as a major cause of gaping and filet softness (Jacobsen et al., 2017). In pigs, heat stress leads to development of pale, soft, exudative (PSE) meat (Strasburg and Chiang, 2009) that is associated with defective Ca^2+^ regulation. Despite a well-developed understanding of meat tenderization that has been studied for decades in mammals, the need exists for genetic markers of the fish “gaping” and filet softness phenotypes (Ouali et al., 2013).

Connective tissue, muscle fiber density, muscle fiber type, postmortem metabolism, and postmortem autolysis are inherent factors affecting muscle texture. Proteolytic degradation of connective tissue, myofibrils, extracellular matrix, and cell membrane constituents contribute to post-mortem softening (Torgersen et al., 2014). Protein content is relatively constant in fish; however, it may vary due to seasonal changes and physiological factors (Delbarre-Ladrat et al., 2006; Belitz et al., 2009). For instance, carbohydrate content and metabolism affect postmortem changes in protein content. Glycolysis determines the rate and extent of pH decline, which affects proteolysis and water-binding ability of the tissue. In turn, proteolysis and water-binding ability influence firmness of porcine muscle (Grześ et al., 2017). However, the pH decline in fish is small due to low glycogen content in the muscle (Belitz et al., 2009). There is general agreement that tenderization is enzymatic in nature and may begin with the onset of apoptosis, followed by proteolysis (Ouali et al., 2013). Enzymatic degradation of key structural proteins that maintain myofibril integrity leads to postmortem tenderization. Calpains, cathepsins, proteasome, and matrix metalloproteases may act in synergy, affected by pH, sarcoplasmic calcium, osmotic pressure, and oxidative processes, to degrade the proteins (Delbarre-Ladrat et al., 2006). The increased level of stress, glycogenolysis, glycolysis, and pH decline (Thomas et al., 2005) in the perimortem period, is associated with increased activity of cathepsin L, which degrades collagen and leads to filet softening. However, protein isoforms of fish may react differently than mammalian species because filet storage temperature are much closer to temperature optimal for proteases, glycolytic enzymes, and pyruvate dehydrogenase to name a few possibilities. Firmness of salmon muscle has been previously attributed to efficient aerobic metabolism and degradation of damaged/misfolded proteins (Torgersen et al., 2014). In addition, atrophying muscle from sexually mature rainbow trout fish showed softer muscle that that of sterile fish (Paneru et al., 2018). Transcriptomic profiling of the atrophying muscle revealed differential expression of genes related to protein ubiquitination, autophagy, extracellular matrix, myofibrillar proteins, and collagen; collectively called “the rainbow trout muscle degradome” (Paneru et al., 2018). Further, profiling muscle transcriptome from fish families exhibiting divergent filet firmness, revealed a network of protein-coding and non-coding genes related to lysosomal and proteolytic activities (Paneru et al., 2017; Ali et al., 2018). Understanding the underlying mechanism of filet firmness will help evaluate the postmortem changes affecting filet quality, and facilitate selective breeding decisions.

Traditional genetic improvement programs to determine animals with elite genetic merit have used statistical analyses of phenotypes and pedigree information (Dang et al., 2014). Genetic selection has been introduced in rainbow trout to improve filet quality (Kause et al., 2007; Hu et al., 2013). Selection programs for fish, including rainbow trout, focused on growth rate and filet quality traits; however, little attention has been paid to filet texture (Bahuaud et al., 2010). Selection on fat content improved color and filet texture (Florence et al., 2015), feed conversion ratio (FCR), and protein-retention efficiency (Kause et al., 2016). Five generations of family based selection was established at the USDA National Center of Cool and Cold Water Aquaculture (NCCCWA) yielding a genetic gain of ∼10% in body weight/ generation (Leeds et al., 2016). Firmness is measured postmortem, thus the trait cannot be measured directly on breeding candidates. Only family specific estimated breeding values (EBVs) are used for breeding candidates in traditional breeding programs. Genomic selection will allow further within-family selection for the filet firmness traits, and thus is anticipated to increase accuracy of genetic predictions and selection response. Understanding the genetic architecture of the filet phenotypic traits and development of genetically improved strains will improve aquaculture industry profitability and consumer satisfaction (Ali et al., 2018).

Genome-wide association (GWA) analysis compares allele frequencies at candidate loci with respect to the studied trait, and takes advantage of linkage disequilibrium (LD) between SNP marker and trait loci (Schielzeth and Husby, 2014). GWA analyses have been extensively used, in mammals including human, to facilitate the investigation of variants association with complex phenotypic traits and diseases (Hindorff et al., 2009). A limited number of GWA analyses have been conducted in fish including Atlantic salmon (Tsai et al., 2015), catfish (Geng et al., 2016), orange-spotted grouper (Yu et al., 2018), and rainbow trout (Gonzalez-Pena et al., 2016; Salem et al., 2018). The studied traits in fish included growth (Tsai et al., 2015; Yu et al., 2018), disease resistance (Palti et al., 2015), head size (Geng et al., 2016), heat stress (Jin et al., 2017), low oxygen tolerance (Zhong et al., 2017), and muscle yield (Gonzalez-Pena et al., 2016; Salem et al., 2018). In rainbow trout, GWA analysis revealed quantitative trait loci (QTL) associated with filet yield and disease resistance (Liu et al., 2015; Palti et al., 2015; Gonzalez-Pena et al., 2016). No GWA studies have been conducted in fish to identify the genetic architecture of filet firmness. However, several GWA studies in cattle and pig revealed some genetic factors affecting meat tenderness. Calpain 1 and calpastatin are among genes that harbored genetic variants associated with meat tenderness in cattle (Ramayo-Caldas et al., 2016).

A 50K transcribed gene SNP chip of average 1 SNP per 42.7 Kb, was recently developed for rainbow trout. About 21K SNPs showing potential association with important traits, including fish growth, muscle yield/quality and filet softness, were used to build the chip. In addition, 29K SNPs were added to the chip following a strategy of 2 SNPs/ gene to randomize the SNP distribution. The recent release of rainbow trout genome (GenBank assembly Accession GCA_002163495, RefSeq assembly accession GCF_002163495) helped in assigning SNPs to chromosomes. Recently, the chip was successfully used to identify several QTL markers associated with muscle yield (Salem et al., 2018). The objective of the current study was to explore the genetic architecture in one of the most important muscle quality attributes, filet firmness in relation to protein content, and identify QTL associated with these traits in a rainbow trout population developed by the USDA/NCCCWA selective breeding program.

## Materials and Methods

### Ethics Statement

Institutional Animal Care and Use Committee of the United States Department of Agriculture, National Center for Cool and Cold Water Aquaculture (Leetown, WV, United States) specifically reviewed and approved all husbandry practices used in this study (IACUC approval #056).

### Fish Population, Tissue Sampling, and Phenotypic Traits

Fish population and tissue sampling were previously described in detail (Al-Tobasei et al., 2017). Briefly, diploid females from a growth-selected line at NCCCWA were used to carry out GWA analysis. This selective breeding program was initiated in 2004 and has gone through 5 generations of selection (Leeds et al., 2016). Third- and fourth-generation fish (Year-class, YC, 2010 and YC 2012) were used for GWA analysis. Phenotypic data were collected from 789 fish representing 98 families from YC 2010 and 99 families from YC 2012. Over a 6-week period, full-sib families were produced from single-sire × single-dam matings. Eggs were reared in spring water and incubated at 7–13°C to hatch all families within a 3-week period. Each family was reared in a separate 200-L tank at ∼12.5°C to retain pedigree information and were fed a commercial fishmeal-based diet (Zeigler Bros Inc., Gardners, PA, United States). At ∼5-months post-hatch, fish were tagged with a passive integrated transponder (Avid Identification Systems Inc., Norco, CA, United States) and reared together in 800-L communal tanks supplied with partially recirculated spring water, at ∼13°C, until ∼13 months post-hatch. Fish were fed a commercial fishmeal-based diet. The feeding schedule was previously described (Hinshaw, 1999). Fish did not receive feed for 5 days prior to harvest to facilitate processing.

Whole body weight (WBW) was measured in fish belonging to each family and families were sorted according to their WBW. The 2nd or 3rd fish from each family was selected for muscle sampling to keep the distribution of WBW consistently adjusted around the median of each family. For each harvest year, selected fish were randomly assigned to one of five harvest groups (∼100 fish each) allowing one fish per family per harvest group. The five groups were sampled in five consecutive weeks (one group/week) each YC. Fish from the YC 2010 were harvested between 410- and 437-days post-hatch (mean body weight = 985 g; *SD* = 239 g), whereas those from YC 2012 were harvested between 446- and 481-days post-hatch (mean body weight = 1,803 g; *SD* = 305 g). Muscle shear force and protein content showed low regression coefficient (*R*^2^) values of 0.05 and 0.04 with body weight, respectively. Fish were euthanized in a lethal dose of tricaine methane sulfonate (Tricaine-S, Western Chemical, Ferndale, WA, United States), harvested, and eviscerated. Head-on gutted carcasses were packed in ice, transported to the West Virginia University Muscle Foods Processing Laboratory (Morgantown, WV, United States), and stored overnight. Carcasses were manually processed into trimmed, skinless filets (Salem et al., 2013). Shear force of 4 × 8 cm section of cooked filet was assessed using a five-blade, Allo-Kramer shear cell attached to a Texture Analyzer (Model TA-HDi^®^; Texture Technologies Corp., Scarsdale, NY, United States), equipped with a 50 kg load cell; tests were performed at a crosshead speed of 127 mm/min (Aussanasuwannakul Kenney et al., 2010). Texture Expert Exceed software (version 2.60; Stable Micro Systems Ltd., Surrey, United Kingdom) was used to record and analyze force-deformation graphs. Peak shear force (g/g sample) was recorded. All cooked texture evaluations were performed approximately 48 h post-harvest. Details of the proximate analyses, including crude protein were previously described (Manor et al., 2015). Crude protein analysis was achieved using AOAC-approved methods (AOAC 2000). Percent Kjeldahl nitrogen (KjeltecTM 2300; Foss North America; Eden Prairie, MN, United States) was converted into crude protein using 6.25 as the conversion factor. The pedigree-based heritability *h^2^* (*h^2^ped*) for protein content and shear force were estimated according to Zaitlen et al. (2013).

### SNP Genotyping and Quality Control

Genotyping was done using a 50K transcribed gene SNP-chip that we recently described and utilized in identifying QTL affecting filet yield (Salem et al., 2018). Source of all SNPs used to build the SNP chip was described in our previous publication (Al-Tobasei et al., 2017). In brief, the array has about 21K SNPs showing potential allelic imbalances with fish body weight, muscle yield, fat content, shear force, whiteness index, and susceptibility to Bacterial Cold Water Disease (BCWD) as we previously described (Al-Tobasei et al., 2017; Salem et al., 2018). In addition, ∼5K non-synonymous SNPs and more SNPs were added to the chip to include at least 2 SNPs per each SNP-harboring gene. The SNP chip includes a total of 50,006 SNPs.

As describe before, a total of 1,728 fish were used to assess quality of this Affymetrix SNP chip. Genotyped fish were obtained from the NCCCWA growth- and BCWD- selection lines (Salem et al., 2018). The SNP chip and sample metrics were calculated. Assessment of quality control (QC) and filtration of samples/genotypes have been performed using the Affymetrix SNPolisher software at the default parameters (Liu et al., 2015). A call rate of 0.97 and Dish QC (DQC) threshold of 0.82 have been applied to filter out genotyped samples. For this study, 789 fish genotyped by the SNP chip had available phenotypic data for filet shear force and protein content. All genotypic data passed the QC. Those fish were used for the current GWA analyses.

### Fifty-SNP Window GWA Analysis

Genome-wide association analysis was performed using the Weighted single-step GBLUP (WssGBLUP) as we previously described (Salem et al., 2018). In brief, WssGBLUP allows use of genotyped and ungenotyped animals. WssGBLUP integrates phenotypic data, genotype and pedigree information in a combined analysis using the following mixed model for single trait analysis:

$$\begin{array}{c} y\;=\;Xb+Z_{1}a+Z_{2}w+e \end{array}$$

Where **y** is the vector of the phenotypes, **b** is the vector of fixed effects including harvest group and hatch year, **a** is the vector of additive direct genetic effects (i.e., animal effect), **w** is the vector of random family effect, and **e** is the residual error. The matrices **X, Z**_1_, and **Z**_2_ are incidence matrices for the effects contained in **b, a**, and **w**, respectively. The model combines all the relationship information (based on pedigree and genotypes) into a single matrix (**H**^-1^).

$$\begin{array}{c} H^{-1}=A^{-1}+\left[\begin{array}{cc} 0 & 0\\ 0 & G^{-1}-A_{22}^{-1} \end{array}\right] \end{array}$$

where **H**^-1^ is the inverse of the realized relationship matrix (**H**), A^-1^ is the inverse of the relationship matrix based on pedigree information, $A_{22}^{-1}$ is the inverse of the pedigree relationship matrix for genotyped animals only, and G^-1^ is the inverse of the genomic relationship matrix. The random family effect is uncorrelated and just accounts for the fact the animals within the same family were raised in a common environment, and the covariance structure is given by I$\sigma_{w}^{2}$, where **I** is an identity matrix and $\sigma_{w}^{2}$ is the family variance.

AIREMLF90 (Misztal et al., 2018) was used to estimate the variance components for the additive direct genetic effect, random family effect, and residuals. The inbreeding value, was previously calculated using a pedigree data of 63,808 fish from five consecutive generations in the NCCCWA breeding program using INBUPGF90 (Misztal et al., 2002; Salem et al., 2018). QC of genomic data was performed using PREGSF90 (Misztal et al., 2014) according to the following settings; MAF > 0.05, call rate > 0.90, and HWE < 0.15. In total, 35,322 SNPs (70.6%) passed QC.

In WssGBLUP analysis, two iterations were used. All SNPs were assigned the same weight during the first iteration (i.e., weight = 1.0). For the second iteration, weights were calculated according to the SNP effects (u^∧^) assessed in the first iteration asu^∧^2p(1 -p), where *p* represents the current allele frequency. Three steps were performed in each iteration: (1) weight was assigned to the SNPs. (2) genomic estimated breeding values (GEBV) were computed using BLUPF90 based on **H**^-1^ (Misztal et al., 2002). (3) SNP effects and weights were calculated using POSTGSF90 (Misztal et al., 2002) based on sliding variance windows of 50 adjacent SNPs. Since the SNPs in the chip were not evenly distributed over the whole genome, the window size used for the current analysis was based on a specific number of adjacent SNPs (*n* = 50 SNPs) instead of physical size (e.g., specific number of nucleotides). A Manhattan plot showing the proportion of additive genetic variance explained by the 50 SNP windows was generated in R using the qqman package (Turner, 2014).

### Single Marker GWA Analysis

Single marker association analysis was conducted using PLINK (Purcell et al., 2007). The phenotypic data were checked for normality using Kolmogorov–Smirnov and Shapiro–Wilk test in order to make sure that the studied phenotypes are normally distributed and meet the assumption of linear model analysis in PLINK (Purcell et al., 2007). For single marker association analysis, the linear model included multiple covariates and accounted for population structure. To control the global inflation of the test statistic, the first five Principal components (PCs) were used as covariates in the model. The Wald test, using the –assoc command, was applied to the quantitative traits in order to retrieve the *R*-squared values of association.

### Gene Annotation and Enrichment Analysis

To retrieve SNP annotations, SNPs bed file was intersected with the rainbow trout genome gff/gtf file using Bedtools as described before (Quinlan and Hall, 2010; Salem et al., 2018). SNPs located within each gene were classified as genic whereas SNPs located outside the body of the gene were classified as intergenic. Genic SNPs were subsequently classified as CDS, intronic, 5′UTR or 3′UTR SNPs. SNPs within long non-coding RNAs (lncRNAs) were determined using a gtf file of our previously published lncRNA reference assembly (Al-Tobasei et al., 2016). SNP-harboring genes were uploaded to the Database for Annotation, Visualization and Integrated Discovery (DAVID) v6.8 (Huang da et al., 2009a,b) to perform gene enrichment analysis (Fisher Exact < 0.05).

## Results and Discussion

**FIGURE 1:**
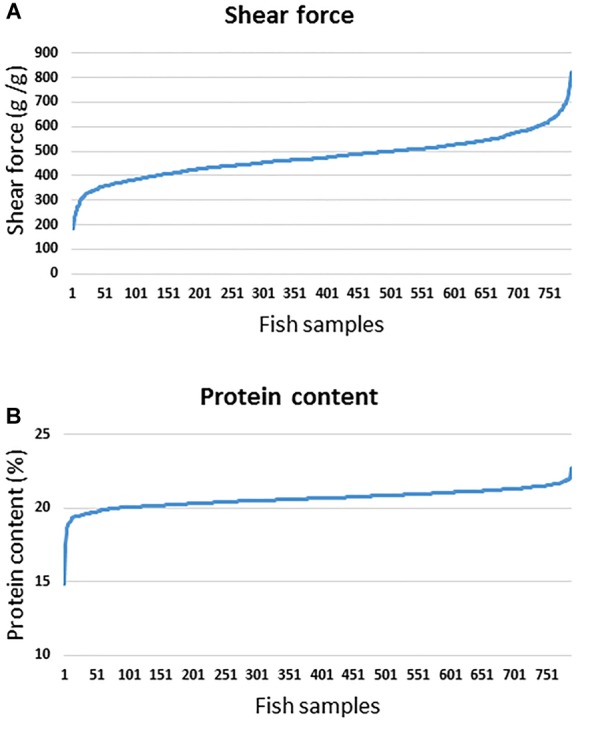
Phenotypic variations in shear force **(A)** and protein content **(B)** among fish samples used for GWA analysis.

Soft flesh is a major criterion for downgrading fish fillets, resulting in loss of value (Michie, 2001). Post-mortem muscle softness is correlated with proteolytic degradation of extracellular matrix and cell membrane components (Bahuaud et al., 2010; Martinez et al., 2011). The fish population used for the current GWA analysis had average shear force of 475.7 ± 83.47 (g/g) and crude protein% = 20.64 ± 0.62. For the current GWA analysis, phenotypic variations in shear force and protein are shown in Figure 1. The estimated heritabilities were 0.33 ± 0.07 and 0.27 ± 0.06 for shear force and protein content, respectively (Zaitlen et al., 2013). Previous studies showed a significant correlation between changes in protein content and meat tenderness (Grześ et al., 2017). Consistently, our data showed a significant correlation between protein content and shear force (*R*^2^ = 0.18; *p*-value < 0.0001). Therefore, we used a 50K SNP chip to perform GWA analyses to identify QTL associated with both traits based on 50 SNP sliding windows using WssGBLUP and single-marker association analyses using PLINK. The chip contains SNPs potentially associated with muscle quality traits including filet softness as we previously described (Al Tobasei et al., 2017; Salem et al., 2018). However, we did not include any fish used in building the SNP-chip for GWA analysis in this study.

### QTL Affecting Filet Shear Force Using WssGBLUP

**FIGURE 2:**
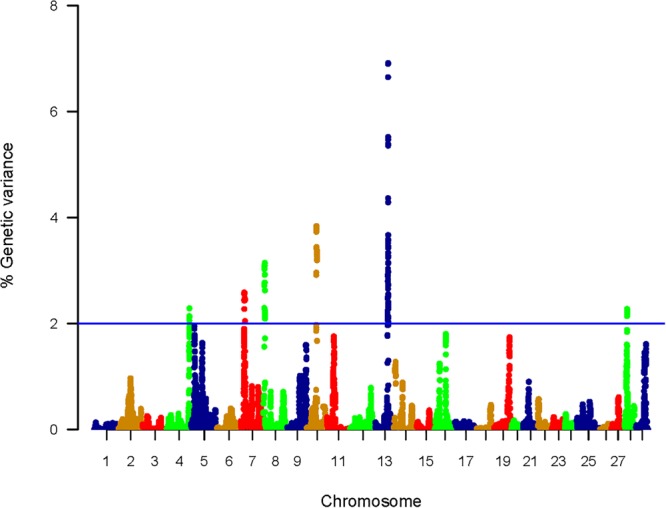
Manhattan plot showing association between genomic sliding windows of 50 SNPs and muscle shear force. Chromosome 13 showed the highest peaks with genomic loci explaining up to 6.91% of the additive genetic variance. The basal blue line represents 2% of additive genetic variance explained by SNPs.

The WssGBLUP-based GWA analysis identified a total of 212 SNPs affecting the additive genetic variance for shear force. These SNPs were located within 95 genes coding for proteins and 4 lncRNAs with 20 SNPs in intergenic regions. SNPs were included in windows explaining at least 2% (arbitrary value) of the additive genetic variance for shear force (Supplementary Table S1). Genomic loci that harbor SNPs were clustered on 6 chromosomes (4, 7, 8, 10, 13, and 28) (Figure 2). Chromosome 13 had the most significant peaks affecting shear force (6.91%) (Supplementary Table S1 and Figure 2) and the highest number of SNPs (*n* = 83) in windows explaining additive genetic variance for shear force (Supplementary Table S1). Many of the SNPs (*n* = 80) were located within the 3′UTR of their genes suggesting a role for these SNPs in microRNA, post-transcriptional regulation of gene expression. Among those 80 SNPs, 32 SNPs created or deleted binding sites for 56 microRNAs (Supplementary Table S2). All QTL associated with genetic variance of shear force are listed in Supplementary Table S1. To gain insights into the biological significance of the identified QTL, we annotated the SNP-harboring genes followed by gene enrichment analysis. Functional annotation showed that SNP-harboring genes were involved in calcium binding/ metabolism, proteolytic activities, apoptotic process, and cellular adhesion and junction (Tables 1, 2). Enriched terms included calcium channel complex, smooth endoplasmic reticulum, ryanodine-sensitive calcium-release channel activity, calcium ion binding, and Z disk (Supplementary Table S3).

**Table 1:** SNP markers in genomic sliding windows of 50 SNPs explaining at least 2% of additive genetic variance in shear force and involved in calcium homeostasis.

Variance		SNP
(%)	CHR	position	Strand	Gene ID	Function	Gene annotation	Region/effect
2.292.29	4	79275235	+	LOC110521100	Calcium metabolism	Ryanodine receptor 3-like	CDS/syn
2.142.14	4	79277177	+	LOC110521100	Calcium metabolism	Ryanodine receptor 3-like	CDS/syn
2.112.11	4	79279144	+	LOC110521100	Calcium metabolism	Ryanodine receptor 3-like	CDS/non-syn
2.062.06	4	79282537	+	LOC110521100	Calcium metabolism	Ryanodine receptor 3-like	Intronic
2.182.18	8	5481101	–	LOC110529177	Calcium metabolism	Ryanodine receptor 3-like	CDS/syn
2.182.18	8	5481500	–	LOC110529177	Calcium metabolism	Ryanodine receptor 3-like	CDS/syn
2.182.18	8	5481770	–	LOC110529177	Calcium metabolism	Ryanodine receptor 3-like	CDS/syn
2.232.23	8	5481890	–	LOC110529177	Calcium metabolism	Ryanodine receptor 3-like	CDS/syn
2.232.23	8	5481962	–	LOC110529177	Calcium metabolism	Ryanodine receptor 3-like	CDS/syn
2.232.23	8	5482326	–	LOC110529177	Calcium metabolism	Ryanodine receptor 3-like	CDS/non-syn
2.232.23	8	5482409	–	LOC110529177	Calcium metabolism	Ryanodine receptor 3-like	CDS/syn
2.232.23	8	5483556	–	LOC110529177	Calcium metabolism	Ryanodine receptor 3-like	CDS/syn
2.232.23	8	5488334	–	LOC110529177	Calcium metabolism	Ryanodine receptor 3-like	CDS/syn
2.242.24	8	5498487	–	LOC110529177	Calcium metabolism	Ryanodine receptor 3-like	CDS/syn
2.242.24	8	5499229	–	LOC110529177	Calcium metabolism	Ryanodine receptor 3-like	CDS/syn
2.192.19	8	5509881	–	LOC110529177	Calcium metabolism	Ryanodine receptor 3-like	CDS/syn
2.192.19	8	5512331	–	LOC110529177	Calcium metabolism	Ryanodine receptor 3-like	CDS/syn
2.922.92	10	33155185	+	LOC110533811	Calcium metabolism	Plastin-3	3′UTR
2.962.96	10	33155312	+	LOC110533811	Calcium metabolism	Plastin-3	3′UTR
3.733.73	10	33155825	+	LOC110533811	Calcium metabolism	Plastin-3	3′UTR
3.753.75	10	33156032	+	LOC110533811	Calcium metabolism	Plastin-3	3′UTR
3.833.83	10	33157280	+	LOC110533811	Calcium metabolism	Plastin-3	3′UTR
3.383.38	10	34861588	–	LOC110533854	Calcium metabolism	TBC1 domain family member 8B-like	3′UTR
3.373.37	10	35666172	–	LOC110533869	Calcium metabolism	Galectin-9-like	CDS/non-syn
3.363.36	10	35668815	–	LOC110533869	Calcium metabolism	Galectin-9-like	CDS/non-syn
5.475.47	13	45250062	–	LOC110486648	Calcium metabolism	Nucleobindin-1-like	3′UTR
6.906.90	13	45250138	–	LOC110486648	Calcium metabolism	Nucleobindin-1-like	3′UTR
5.525.52	13	45348326	+	LOC110486653	Calcium metabolism	Myosin-binding protein C, fast-type-like	CDS/syn
4.294.29	13	45348905	+	LOC110486653	Calcium metabolism	Myosin-binding protein C, fast-type-like	CDS/syn
3.593.59	13	45353098	+	LOC110486653	Calcium metabolism	Myosin-binding protein C, fast-type-like	Intronic
3.573.57	13	45358893	+	LOC110486653	Calcium metabolism	Myosin-binding protein C, fast-type-like	CDS/non-syn
3.673.67	13	45494621	–	LOC110486657	Calcium metabolism	Protein kinase C and casein kinase substrate in neurons protein 3-like	3′UTR
3.133.13	13	45495127	–	LOC110486657	Calcium metabolism	Protein kinase C and casein kinase substrate in neurons protein 3-like	3′UTR
3.253.25	13	45495294	–	LOC110486657	Calcium metabolism	Protein kinase C and casein kinase substrate in neurons protein 3-like	3′UTR
3.283.28	13	45497545	–	LOC110486657	Calcium metabolism	Protein kinase C and casein kinase substrate in neurons protein 3-like	CDS/non-syn
2.982.98	13	45641799	–	LOC110486661	Calcium metabolism	Coronin-1A-like	3′UTR
2.742.74	13	45644102	–	LOC110486661	Calcium metabolism	Coronin-1A-like	CDS/syn
2.352.35	13	45825907	+	LOC110486680	Calcium metabolism	Myosin regulatory light chain 2, skeletal muscle isoform-like	Intronic
2.362.36	13	45826199	+	LOC110486680	Calcium metabolism	Myosin regulatory light chain 2, skeletal muscle isoform-like	3′UTR
2.512.51	13	45826267	+	LOC110486680	Calcium metabolism	Myosin regulatory light chain 2, skeletal muscle isoform-like	3′UTR
2.252.25	28	9763838	–	LOC110508483	Calcium metabolism	Matrix metalloproteinase-14-like	3′UTR
2.262.26	28	9763927	–	LOC110508483	Calcium metabolism	Matrix metalloproteinase-14-like	3′UTR
2.262.26	28	9764282	–	LOC110508483	Calcium metabolism	Matrix metalloproteinase-14-like	3′UTR
2.272.27	28	9764561	–	LOC110508483	Calcium metabolism	Matrix metalloproteinase-14-like	3′UTR
2.182.18	28	9767473	–	LOC110508483	Calcium metabolism	Matrix metalloproteinase-14-like	CDS/syn
2.142.14	28	9771512	–	LOC110508483	Calcium metabolism	Matrix metalloproteinase-14-like	CDS/syn
2.212.21	28	9784531	–	LOC110508483	Calcium metabolism	Matrix metalloproteinase-14-like	5′UTR

*A color gradient on the left indicates differences in additive genetic variance explained by windows containing the representative SNP marker (green is the highest and red is the lowest). SNPs are sorted according to their chromosome positions.*

**Table 2:** SNP markers in genomic sliding windows of 50 SNPs explaining at least 2% of additive genetic variance in shear force and involved in proteolytic, apoptotic process, tight junction, and focal adhesion.

Variance		SNP
(%)	CHR	position	Strand	Gene ID	Function	Gene annotation	Region/effect
2.472.47	7	14194291	–	LOC110527456	Tight junction	Actin-related protein 3	CDS/syn
3.053.05	8	3673516	+	LOC110529290	Autophagy	Zinc finger FYVE domain-containing protein 1-like	CDS/syn
3.053.05	8	3673957	+	LOC110529290	Autophagy	Zinc finger FYVE domain-containing protein 1-like	3′UTR
3.833.83	10	33231241	–	LOC110533814	Endosome	Charged multivesicular body protein 1b	CDS/syn
3.443.44	10	34812751	+	LOC110533848	Autophagy	Immunoglobulin-binding protein 1-like	CDS/syn
3.373.37	10	35666172	–	LOC110533869	Apoptosis	Galectin-9-like	CDS/non-syn
3.363.36	10	35668815	–	LOC110533869	Apoptosis	galectin-9-like	CDS/non-syn
2.232.23	13	43044831	+	LOC110485193	Tight junction	Na(+)/H(+) exchange regulatory cofactor NHE-RF1-like	3′UTR
3.523.52	13	45087094	+	LOC110486632	Lysosome	Granulins-like	CDS/syn
4.364.36	13	45089988	+	LOC110486632	Lysosome	Granulins-like	3′UTR
5.385.38	13	45111046	–	LOC110486641	Amino acid catabolism	Branched-chain-amino-acid aminotransferase, cytosolic-like	3′UTR
5.365.36	13	45142682	+	LOC110486644	Proteolysis	Potassium voltage-gated channel subfamily A member 1-like	CDS/syn
5.475.47	13	45284153	–	LOC110486651	Apoptosis	Tripartite motif-containing protein 16-like	3′UTR
3.463.46	13	45619953	–	LOC110486660	Focal adhesion	Serine/threonine-protein phosphatase alpha-2 isoform-like	3′UTR
3.153.15	13	45620629	–	LOC110486660	Focal adhesion	Serine/threonine-protein phosphatase alpha-2 isoform-like	3′UTR
2.982.98	13	45641167	+	LOC110486662	Tight junction	Claudin-4-like	3′UTR
2.982.98	13	45641799	–	LOC110486661	Phagosome	Coronin-1A-like	3′UTR
2.742.74	13	45644102	–	LOC110486661	Phagosome	Coronin-1A-like	CDS/syn
2.352.35	13	45825907	+	LOC110486680	Focal adhesion	Myosin regulatory light chain 2, skeletal muscle isoform-like	Intronic
2.362.36	13	45826199	+	LOC110486680	Focal adhesion	Myosin regulatory light chain 2, skeletal muscle isoform-like	3′UTR
2.512.51	13	45826267	+	LOC110486680	Focal adhesion	Myosin regulatory light chain 2, skeletal muscle isoform-like	3′UTR
2.232.23	28	9751858	+	LOC110508482	Apoptosis	Apoptotic chromatin condensation inducer in the nucleus-like	CDS/non-syn
2.232.23	28	9753870	+	LOC110508482	Apoptosis	Apoptotic chromatin condensation inducer in the nucleus-like	CDS/syn
2.232.23	28	9754864	+	LOC110508482	Apoptosis	Apoptotic chromatin condensation inducer in the nucleus-like	3′UTR
2.272.27	28	9754973	+	LOC110508482	Apoptosis	Apoptotic chromatin condensation inducer in the nucleus-like	3′UTR
2.252.25	28	9843505	+	LOC110508486	Cell adhesion	Cerebellin-1-like	3′UTR

*A color gradient on the left indicates differences in additive genetic variance explained by windows containing the representative SNP marker (green is the highest and red is the lowest). SNPs are sorted according to their chromosome positions.*

#### SNPs in Genes Affecting Ca^2+^ Homeostasis

Ten genes necessary for calcium metabolism harbored 47 SNPs affecting the genetic variation in shear force (Table 1). Ryanodine receptor3 (RYR3; member of the sarcoplasmic reticulum calcium release channel) had 17 SNPs located on chromosome 4 and 8 suggesting an important role for calcium in regulating shear force.

Two SNPs were non-synonymous, and one of these SNPs exists in the third structural repeat that is conserved in all RYR isoforms; it is located in the N-terminal part of the cytoplasmic region of the RYRs. Several studies reported a correlation between development of pale, soft and exudative (PSE) meat and abnormality in calcium release mechanism of porcine skeletal muscle as a result of a point mutation in the porcine RYR1 that led to a substitution of cysteine for arginine (Arg615Cys) (Fujii et al., 1991). Poor regulation of the mutant channel led to accumulation of sarcoplasmic calcium and development of PSE meat accordingly (MacLennan and Phillips, 1992). Breeding strategies were initiated to avoid this mutation from the pig populations.

Unlike in mammals where RYR1 is the main isoform expressed in skeletal muscle, fish co-express RYR3 (Murayama and Kurebayashi, 2011). Absence of RYR1 in fish, causes slow swimming, weak contractions and reduced Ca^2+^ transients (Hirata et al., 2007). On the other hand, RYR3 knock-down led to reduction in formation of anatomical structures called the parajunctional feet (PJF), which are located on the sides of the SR junctional cisternae (Perni et al., 2015). Reduction of the PJF was coupled with reduced SR Ca^2+^ flux that causes Ca^2+^ sparks that was reported in fish muscle. However, the muscle fibers looked structurally normal and the swimming behavior was not affected (Perni et al., 2015). Association of RYR1&3 mRNA expression level with filet water holding capacity was reported in the Nile tilapia under pre-slaughter stress (Goes et al., 2015). Impaired Ca^2+^ handling was reported in the muscles of the hatchery-reared salmon compared to that of wild fish (Anttila et al., 2008). Levels of RYR were greatly reduced in the muscles of the hatchery-reared salmon. Similar differences were seen in the oxidative capacity of muscles. This impairment was suggested to contribute to the lower swimming capacity of the reared fish (Anttila et al., 2008).

Chromosome 13 had 15 SNPs in Ca^2+^ homeostasis-relevant genes located in top windows affecting the genetic variability in shear force (Table 1). Nucleobindin 1, is a multidomain calcium-binding protein of unclear physiological and biochemical functions (Kapoor et al., 2010) and harbored 2 SNPs within the 3′UTR representing the highest peak in this QTL. The second gene on this chromosome, myosin-binding protein C, fast (MyBP-C), encompassed 4 SNPs. MyBP-C sensitizes the actin thin filaments to Ca^2+^ (Lin et al., 2018). MyBPC gene knockout in mice leads to muscle hypertrophy and impaired contractile function. The third gene, protein kinase C and casein kinase substrate in neurons protein 3 (PACSIN 3) had 4 SNPs. PACSIN 3 has been primarily identified in muscle and lung (Bai et al., 2012). PACSIN 3 is known to modulate the subcellular localization of TRPV4 (Cuajungco et al., 2006) which regulates Ca^2+^ homeostasis and cytoskeletal remodeling (Ryskamp et al., 2016). Coronin-1 represents the fourth gene and had two SNPs. It mediates Ca^2+^ mobilization from the intracellular stores (Mueller et al., 2008). The fifth gene, myosin regulatory light chain 2 (MYL2), had three SNPs within 3′UTR. MYL2 is a calcium-binding chain known to be associated with meat tenderness (Rosa et al., 2018).

Eight SNPs were also identified in 3 genes necessary for calcium metabolism on chromosome 10 (Table 1). Plastin-3 (PLS3) had five SNPs in windows explaining up to 3.83% of the additive genetic variance in shear force. PLS3 functions as a protective modifier of spinal muscular atrophy in Ca^+2^-dependent manner (Lyon et al., 2014). A single SNP was identified in a gene that codes for TBC1 domain family member 8B (TBC1D8) and has GO terms belong to calcium ion binding. Galectin-9 (Gal-9) harbored two SNPs within windows explaining ∼3.37% of the additive genetic variance in shear force. Gal-9 induces apoptosis via the Ca^2+^-calpain-caspase-1 pathway (Kashio et al., 2003).

Chromosome 28 had a single gene, matrix metalloproteinase-14 (MMP14), that had 7 SNPs explaining at least 2.0% of the additive genetic variance (Table 1). MMP14 has a Ca^2+^-dependent catalytic MP domain that degrades the extracellular matrix proteins such as collagen (Tallant et al., 2010). Our recent studies showed that MMP9 was downregulated in trout families of high shear force suggesting a role for matrix metalloproteinase family in regulating filet firmness in fish (Ali et al., 2018). In addition, transcripts of stanniocalcin (STC), the main regulatory hormone of Ca^2+^ homeostasis in fish (Verma and Alim, 2014), were overexpressed in trout families with high shear force (Ali et al., 2018). The relationship between calcium and protein content in dystrophic muscle has been attributed to decreased functionality of the sarcoplasmic reticulum to sequester calcium ions (Kameyama and Etlinger, 1979). Together, our results indicate a major role of Ca^2+^ homeostasis in determining fish filet firmness.

#### SNPs in Genes Affecting Proteolysis

Six SNP-harboring genes involved in proteolytic/ catabolic and apoptotic processes were identified on chromosomes 10, 13, and 28 (Table 2). Chromosome 10 had a gene that codes for Gal-9 which is known to induce apoptotic process (Kashio et al., 2003). Chromosome 13 had four genes harboring SNPs within top windows affecting the additive genetic variance in shear force. First, tripartite motif-containing protein 16, affecting 5.47% of the additive genetic variance, promotes apoptosis by modulating the caspase-2 activity. Second, branched-chain-amino-acid aminotransferase (cytosolic), had a single 3′UTR SNP. This enzyme catalyzes the first reaction in the catabolism of the most hydrophobic branched chain amino acids (leucine, isoleucine, and valine) that play important roles in determining the structure of globular proteins, in addition to the interaction of transmembrane domains with the phospholipid layer (Blomstrand et al., 2006). Third, potassium voltage-gated channel, subfamily A member 1 harbored a single synonymous SNP. Voltage-dependent potassium channels mediates transmembrane potassium transport and are involved in the proteolytic system that causes postmortem tenderization (Mateescu et al., 2017). The fourth gene in the list codes for granulins that had 2 SNPs. Granulins have possible critical lysosomal functions, and their loss is an initiating factor in lysosomal dysfunction (Holler et al., 2017). In addition, chromosome 28 had four SNPs in a gene coding for apoptotic chromatin condensation inducer in the nucleus (ACIN1) (Table 2). ACIN1 belongs to the prominent canonical apoptosis signaling pathway (Schrötter et al., 2012).

Two SNP-harboring genes were mapped to the autophagy pathway; immunoglobulin-binding protein 1 (IGBP1) and zinc finger FYVE domain-containing protein 1 (ZFYVE1). ZFYVE1, has been used as a marker of omegasomes (exists only during autophagosome formation) (Zientara-Rytter and Subramani, 2016). Three SNPs spanning two genes coding for coronin-1A and charged multivesicular body protein 1b, were mapped to the endosomal/phagosomal pathway. Previous studies support presence of phagocytic activities in postmortem muscle to eliminate extracellular material (Ouali et al., 2013).

#### SNPs in Genes Affecting Cell Adhesion

Genes involved in focal adhesion and cell junction were previously reported to be associated with meat tenderness (Fonseca et al., 2017). Five SNPs spanning two genes on chromosome 13 were mapped to the focal adhesion pathway (Table 2). These genes code for myosin regulatory light chain 2 and serine/threonine-protein phosphatase alpha-2. In addition, 2 SNPs were identified spanning two genes involved in tight junction pathway (Table 2). The two genes are located on chromosomes 7 and 13, and code for Na (+)/H (+) exchange regulatory cofactor NHE-RF1 and actin-related protein 3. Cerebellin-1 on chromosome 28, had a single SNP in a window explaining 2.25% of the additive genetic variance (Table 2). Functional annotation analysis showed that cerebellin-1 has GO terms belonging to heterophilic, cell-cell adhesion via plasma membrane, cell adhesion molecules. The list also includes a SNP in a gene on chromosome 13, that codes for claudin-4 (Table 2). This SNP creates a binding site for the mir-10c-5p. mir-10c-5p showed differential expression association with shear force in trout fish families of YC 2010 (Paneru et al., 2017). Members of the claudins family are major integral membrane proteins existing at tight junctions, and they have Ca^2+^-independent cell-adhesion activity (Kubota et al., 1999).

### QTL Affecting Protein Content Using WssGBLUP

**FIGURE 3:**
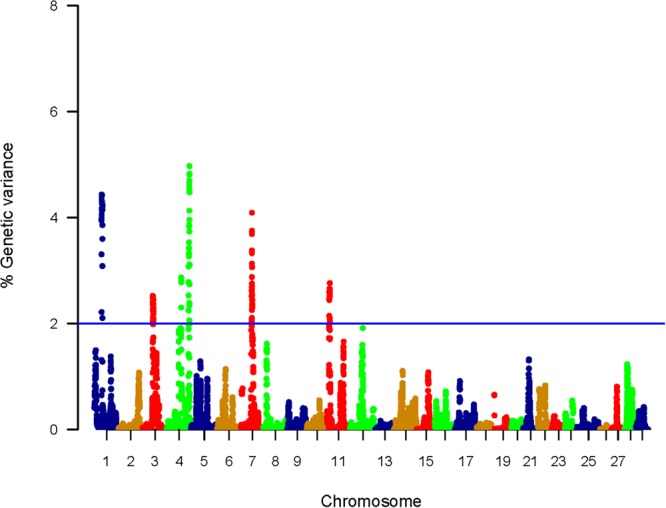
Manhattan plot showing association between genomic sliding windows of 50 SNPs and muscle protein content. Chromosome 4 showed the highest peaks with genomic loci explaining up to 4.97% of the additive genetic variance. The basal blue line represents 2% of additive genetic variance explained by SNPs.

In total, 225 SNPs affecting the genetic variation in muscle protein content were identified; 202 genic and 23 intergenic SNPs (Supplementary Table S4). Each SNP was in a window explaining at least 2% of the additive genetic variance for the protein content. The genomic loci that harbor SNPs were clustered on five chromosomes (1, 3, 4, 7, and 11) (Figure 3). Chromosomes 4 and 1 harbored 50 SNPs located within top windows affecting the genetic variability (variance > 4.0%) in protein content of the muscle (Supplementary Table S4). Similar to shear force, 40% of the SNPs were located within 3′UTR. Thirteen SNPs created/ deleted target sites for 16 microRNAs (Supplementary Table S5). SNPs associated with genetic variation in crude protein content are listed in Supplementary Table S4. Functional annotation followed by gene enrichment analysis were performed to functionally characterize the SNP-harboring genes. Functional annotation showed that SNP-harboring genes were mainly involved in apoptotic process, proteolysis, lysosomal activities, cell proliferation, transcription, and methylation (Tables 3, 4). Enriched terms included muscle contraction, transcription, regulation of transcription, and chromatin remodeling (Supplementary Table S6).

**Table 3:** SNP markers in genomic sliding windows of 50 SNPs explaining at least 2% of additive genetic variance in protein content and involved in proteolytic and apoptotic processes.

Variance		SNP
(%)	CHR	position	Strand	Gene ID	Function	Gene annotation	Region/effect
2.222.22	1	25420470	+	LOC110520559	Proteolysis	Aspartate aminotransferase, mitochondrial-like	3′UTR
2.022.02	3	35652142	–	LOC110518458	Proteolysis	Plectin-like	CDS/syn
2.412.41	3	35653189	–	LOC110518458	Proteolysis	Plectin-like	CDS/syn
2.362.36	3	35653735	–	LOC110518458	Proteolysis	Plectin-like	CDS/syn
2.492.49	3	35654687	–	LOC110518458	Proteolysis	Plectin-like	CDS/non-syn
2.492.49	3	35654724	–	LOC110518458	Proteolysis	Plectin-like	CDS/non-syn
2.522.52	3	35654840	–	LOC110518458	Proteolysis	Plectin-like	CDS/non-syn
2.512.51	3	35654914	–	LOC110518458	Proteolysis	Plectin-like	CDS/syn
2.512.51	3	35657920	–	LOC110518458	Proteolysis	Plectin-like	CDS/syn
2.522.52	3	35673163	–	LOC110518458	Proteolysis	Plectin-like	CDS/syn
2.042.04	3	36517107	+	LOC110518871	Proteolysis	2-oxoisovalerate dehydrogenase subunit beta, mitochondrial-like	3′UTR
2.052.05	3	36517385	+	LOC110518871	Proteolysis	2-oxoisovalerate dehydrogenase subunit beta, mitochondrial-like	3′UTR
2.182.18	3	36865549	–	LOC110519010	Proteolysis	Inactive serine protease 35-like	CDS/non-syn
2.302.30	4	50656794	–	LOC110522013	Proteolysis and apoptosis	Rho-related GTP-binding protein RhoB	3′UTR
2.872.87	4	50657566	–	LOC110522013	Proteolysis and apoptosis	Rho-related GTP-binding protein RhoB	CDS/syn
2.792.79	4	50753202	+	LOC110522012	Proteolysis	Lysosomal-associated transmembrane protein 4A-like	3′UTR
2.782.78	4	50753210	+	LOC110522012	Proteolysis	Lysosomal-associated transmembrane protein 4A-like	3′UTR
2.782.78	4	50753431	+	LOC110522012	Proteolysis	Lysosomal-associated transmembrane protein 4A-like	3′UTR
3.303.30	4	77336642	–	LOC110522556	Proteolysis	V-type proton ATPase subunit D-like	3′UTR
3.293.29	4	77336859	–	LOC110522556	Proteolysis	V-type proton ATPase subunit D-like	3′UTR
3.293.29	4	77338088	–	LOC110522S56	Proteolysis	V-type proton ATPase subunit D-like	CDS/syn
3.293.29	4	77342367	–	LOC110522556	Proteolysis	V-type proton ATPase subunit D-like	CDS/syn
3.293.29	4	77347822	–	LOC110522557	Apoptosis	RNA-binding protein 25-like	CDS/syn
3.303.30	4	77352343	–	LOC110522557	Apoptosis	RNA-binding protein 25-like	CDS/non-syn
3.313.31	4	77453100	–	LOC110522560	Proteolysis	26S protease regulatory subunit 4-like	CDS/syn
3.423.42	4	78252725	+	LOC110522566	Apoptosis	SNW domain-containing protein 1-like	CDS/syn
3.423.42	4	78252740	+	LOC110522S66	Apoptosis	SNW domain-containing protein 1-like	CDS/non-syn
3.423.42	4	78254203	+	LOC110522566	Apoptosis	SNW domain-containing protein 1-like	CDS/syn
3.533.53	4	78263097	+	LOC110522566	Apoptosis	SNW domain-containing protein 1-like	3′UTR
4.624.62	4	79069732	+	LOC110522582	Apoptosis	Actin, alpha cardiac	CDS/syn
4.624.62	4	79071472	+	LOC110522582	Apoptosis	Actin, alpha cardiac	CDS/syn
2.532.53	7	38918677	–	LOC110527901	Apoptosis	bcl-2-like protein 1	3′UTR
2.752.75	7	39093852	+	LOC110527910	Apoptosis	Protein snail homolog Sna-like	3′UTR
3.383.38	7	39160373	–	LOC110527912	Proteolysis	Short transient receptor potential channel 4-associated protein-like	CDS/syn
2.302.30	7	40277695	+	LOC110527935	Apoptosis	Cell death activator CIDE-3-like	3′UTR
2.572.57	11	9183364	–	LOC110535270	Proteolysis	NEDD8 ultimate buster 1-like	CDS/syn

*A color gradient on the left indicates differences in additive genetic variance explained by windows containing the representative SNP marker (green is the highest and red is the lowest). SNPs are sorted according to their chromosome positions.*

**Table 4:** SNP markers in genomic sliding windows of 50 SNPs explaining at least 2% of genetic variance in protein content and involved in calcium metabolism, cell proliferation, and transcriptional/ chromatin regulations.

Variance		SNP
(%)	CHR	position	Strand	Gene ID	Function	Gene annotation	Region/effect
3.973.97	1	25480866	–	LOC110520608	Chromatin regulator	Histone-lysine *N*-methyltransferase KMT5B-like	CDS/syn
4.434.43	1	25956429	+	LOC110520691	Proliferation	Myocyte-specific enhancer factor 2A-like	3′UTR
4.424.42	1	25956638	+	LOC110520691	Proliferation	Myocyte-specific enhancer factor 2A-like	3′UTR
4.204.20	1	28282718	+	rcn2	Calcium and proliferation	Reticulocalbin 2	CDS/syn
2.512.51	3	36206239	+	LOC110518743	Chromatin regulator	Transcription and mRNA export factor ENY2-2	3’UTR
2.512.51	3	36206278	+	LOC110518743	Chromatin regulator	Transcription and mRNA export factor ENY2-2	3’UTR
2.102.10	3	36651473	–	LOC110518895	Calcium metabolism	Inhibitor of Bruton tyrosine kinase-like	3’UTR
2.112.11	3	36662440	–	LOC110518895	Calcium metabolism	Inhibitor of Bruton tyrosine kinase-like	CDS/non-syn
2.092.09	3	36662456	–	LOC110518895	Calcium metabolism	Inhibitor of Bruton tyrosine kinase-like	CDS/non-syn
2.092.09	3	36672488	–	LOC110518895	Calcium metabolism	Inhibitor of Bruton tyrosine kinase-like	CDS/syn
2.172.17	3	37260197	+	LOC110519152	Chromatin regulator	Ubiquinone biosynthesis O-methyltransferase, mitochondrial-like	CDS/syn
2.192.19	3	37276563	+	LOC110519152	Chromatin regulator	Ubiquinone biosynthesis O-methyltransferase, mitochondrial-like	3’UTR
2.092.09	3	37664570	+	LOC110519270	Calcium metabolism	Protein FAM26E-like	CDS/non-syn
2.782.78	4	76616397	+	LOC110522552	Chromatin regulator	Ribosomal oxygenase l-like	3’UTR
3.273.27	4	77424809	–	LOC110522561	Calcium metabolism	Calmodulin	3’UTR
3.303.30	4	77425427	–	LOC110522561	Calcium metabolism	Calmodulin	3’UTR
3.303.30	4	77425526	–	LOC110522561	Calcium metabolism	Calmodulin	3’UTR
3.423.42	4	78252725	+	LOC110522566	Chromatin regulator	SNW domain-containing protein 1-like	CDS/syn
3.423.42	4	78252740	+	LOC110522566	Chromatin regulator	SNW domain-containing protein 1-like	CDS/non-syn
3.423.42	4	78254203	+	LOC110522566	Chromatin regulator	SNW domain-containing protein 1-like	CDS/syn
3.533.53	4	78263097	+	LOC110522566	Chromatin regulator	SNW domain-containing protein 1-like	3’UTR
3.863.86	4	79017410	–	LOC110522579	Transcription	Poly(A) polymerase beta-like	3’UTR
3.853.85	4	79017997	–	LOC110522579	Transcription	Poly(A) polymerase beta-like	3’UTR
4.564.56	4	79019016	–	LOC110522579	Transcription	Poly(A) polymerase beta-like	3’UTR
4.634.63	4	79039957	–	LOC110522579	Transcription	Poly(A) polymerase beta-like	CDS/syn
4.694.69	4	79274809	+	LOC110521100	Calcium metabolism	Ryanodine receptor 3-like	CDS/syn
4.814.81	4	79275235	+	LOC110521100	Calcium metabolism	Ryanodine receptor 3-like	CDS/syn
4.814.81	4	79277177	+	LOC110521100	Calcium metabolism	Ryanodine receptor 3-like	CDS/syn
4.824.82	4	79279144	+	LOC110521100	Calcium metabolism	Ryanodine receptor 3-like	CDS/non-syn
4.974.97	4	79282537	+	LOC110521100	Calcium metabolism	Ryanodine receptor 3-like	Intronic
4.134.13	4	79284060	+	LOC110521100	Calcium metabolism	Ryanodine receptor 3-like	CDS/syn
4.514.51	4	79285101	+	LOC110521100	Calcium metabolism	Ryanodine receptor 3-like	CDS/syn
4.544.54	4	79307585	+	LOC110521100	Calcium metabolism	Ryanodine receptor 3-like	CDS/syn
4.484.48	4	79320253	+	LOC110521100	Calcium metabolism	Ryanodine receptor 3-like	CDS/syn
3.963.96	4	79320321	+	LOC110521100	Calcium metabolism	Ryanodine receptor 3-like	CDS/syn
3.743.74	4	79322346	+	LOC110521100	Calcium metabolism	Ryanodine receptor 3-like	CDS/syn
3.113.11	4	79330676	+	LOC110521100	Calcium metabolism	Ryanodine receptor 3-like	CDS/non-syn
3.093.09	4	79330715	+	LOC110521100	Calcium metabolism	Ryanodine receptor 3-like	CDS/non-syn
2.742.74	4	79331299	+	LOC110521100	Calcium metabolism	Ryanodine receptor 3-like	CDS/non-syn
2.742.74	4	79335498	+	LOC110521100	Calcium metabolism	Ryanodine receptor 3-like	CDS/non-syn
2.392.39	4	79336796	+	LOC110521100	Calcium metabolism	Ryanodine receptor 3-like	CDS/syn
2.342.34	4	79347211	+	LOC110521100	Calcium metabolism	Ryanodine receptor 3-like	CDS/syn
2.052.05	4	79348423	+	LOC110521100	Calcium metabolism	Ryanodine receptor 3-like	CDS/syn
2.052.05	4	79349026	+	LOC110521100	Calcium metabolism	Ryanodine receptor 3-like	CDS/syn
2.512.51	7	38897108	+	LOC110527899	Chromatin regulator	Host cell factor 1-like	3’UTR
2.512.51	7	38897671	+	LOC110527899	Chromatin regulator	Host cell factor 1-like	3’UTR

*A color gradient on the left indicates differences in additive genetic variance explained by windows containing the representative SNP marker (green is the highest and red is the lowest). SNPs are sorted according to their chromosome positions.*

#### SNPs in Genes Affecting Apoptosis

Thirteen SNPs were identified spanning seven genes on chromosomes 4 and 7, and engaged in apoptotic process (Table 3). Actin, alpha harbored two SNPs in windows that explained the highest genetic variability (4.62%) in this category. Alpha actin was previously suggested as a genetic marker for apoptosis (Ouali et al., 2013). SNW domain-containing protein 1 (SNW1) harbored 4 SNPs in windows explaining up to 3.53% of the additive genetic variance. Depletion of SNW1 or its associating proteins induced apoptotic processes in cancer cells (Sato et al., 2015). Three SNPs were identified in RNA-binding protein 25 (RBM25) and Bcl-2-like protein 1 (BCL2L1). RBM25 is involved in apoptotic cell death by regulating BCL2L1 expression (Zhou et al., 2008). Two SNPs were identified in RHOB that is known to positively regulate apoptotic process (Srougi and Burridge, 2011). A single 3′UTR SNP was identified in a gene coding for protein snail homolog Snai. Snai1-expressing cells resists apoptosis triggered by proapoptotic stimuli (Olmeda et al., 2007). Another 3′UTR SNP was also identified in a gene coding for cell death activator CIDE-3. This gene has a role in the execution phase of apoptosis (Liang et al., 2003).

#### SNPs in Genes Affecting Proteolysis

Ten genes with proteolytic activities were identified that affected genetic variability in protein content (Table 3). A single SNP located in the gene coding for short transient receptor potential channel 4-associated protein (TRPC4AP) followed by a SNP in 26S protease regulatory subunit 4 (PSMC1) came at the top of this group. TRPC4AP is involved in ubiquitination and destruction of Myc protein (Choi et al., 2010) that control cell proliferation and growth (Bernard and Eilers, 2006). Whereas, PSMC1 is a component of the 26S proteasome that maintains protein homeostasis through ubiquitin-mediated degradation of damaged and misfolded proteins (Kanayama et al., 1992). NEDD8 ultimate buster 1 (NUB1) and inactive serine protease 35 (PRSS35) had a single SNP. NUB1 positively regulates proteasomal ubiquitin-dependent protein catabolic process (Schmidtke et al., 2006) whereas, the proteolytic activities of the serine protease, PRSS35, have not been characterized yet (Diao et al., 2013). Plectin had nine SNPs. In human, mutations of the plectin gene cause muscular dystrophy (Natsuga et al., 2010). The list also includes two mitochondrial genes, encoding for 2-oxoisovalerate dehydrogenase subunit beta and aspartate aminotransferase, involved in amino acid catabolism (Schiele et al., 1989; Nobukuni et al., 1991). Of note, three genes on chromosome 4 were involved in lysosomal activities; V-type proton ATPase subunit D, Rho-related GTP-binding protein RhoB (RHOB), and lysosomal-associated transmembrane protein 4A (LAPTM4A). V-type proton ATPase subunit D had 4 SNPs in windows explaining up to 3.30% of the genetic variation in crude protein content. The vacuolar (H^+^)-ATPases acidify the intracellular compartments and play an important role in protein degradation (Toei et al., 2010). RHOB is involved in trafficking epidermal growth factor (EGF) receptor from late endosomes to lysosomes (Gampel et al., 1999). Three SNPs were identified in LAPTM4A. The function of this gene is unclear.

#### SNPs in Genes Affecting Ca^2+^ Homeostasis

We identified 28 SNPs, within 5 genes on chromosomes 1, 3, and 4, that are involved in calcium homeostasis (Table 4). Interestingly, RYR3 harbored ∼ 68% of those SNPs; whereas four of these SNPs affected genetic variability in shear force. This result suggests a major role for RYR3 in regulating protein content and shear force in rainbow trout. SNPs of RYR3 were ranked first in this category and were located within windows explaining up to 4.97% of the additive genetic variance in protein content. A single SNP was identified within a gene that codes for reticulocalbin 2 (RCN2). Previous studies showed that RCN2 binds to calcium and was identified to be localized in endoplasmic reticulum. RCN2 was upregulated in hepatocellular carcinoma patients and its homozygous deletion in mice was lethal (Wang et al., 2017). In addition, there were three 3′UTR SNPs within the calmodulin (CaM) gene. CaM codes for a calcium binding protein known to regulate RYR activity through direct binding to a CaM-binding domain of RYR (Oo et al., 2015). In addition, two genes coding for inhibitor of Bruton tyrosine kinase (Btk) and protein FAM26E (CALHM1) harbored 5 SNPs on chromosome 3. Btk plays a role in releasing sequestered Ca^2+^ to the cytosol (Liu et al., 2001). Whereas, CALHM1 detects the extracellular Ca^2+^ level and plays a role in Ca^2+^ homeostasis (Ma et al., 2012). These results suggest a significant role of the genes involved in Ca^2+^ handling (release and re-sequestration). In mammals, proteolysis by calcium-dependent proteases (calpains) in the early postmortem period greatly affects muscle texture and meat tenderization (Koohmaraie, 1992; Duckett et al., 2000). We previously showed that calpains are elevated and calpastatin is reduced during starvation-induced muscle degradation (Salem et al., 2005a, 2007) and calpastatin expression is associated with rainbow trout muscle growth (Salem et al., 2005b). Further studies are warranted to investigate postmortem autolysis caused by calpain system in regulating protein content and shear force in rainbow trout.

#### SNPs in Genes Affecting Transcriptional Process and Cell Proliferation

Genes involved in transcription and cell proliferation were identified (Table 4). The majority of SNP-harboring genes were involved in transcription. Sixty-six SNPs were identified in 26 genes located, mainly, on chromosomes 4 and 7. Four SNPs in a gene that code for poly(A) polymerase beta were identified in windows explaining the highest genetic variability (4.63%) in this category.

Additionally, twelve SNPs located on six genes involved in cell proliferation were identified. Three SNPs on two genes that code for myocyte-specific enhancer factor 2A (MEF2) and RCN2 were ranked at the top of this group. MEF2 plays diverse roles in muscle to control myogenesis (Black and Olson, 1998).

#### SNPs in Genes Affecting Histone Modifications

Twelve SNPs in six genes involved in epigenetic transcriptional regulation were also identified on chromosomes 1, 3, 4, and 7 (Table 4). Histone-lysine *N*-methyltransferase KMT5B (KMT5B) had a single SNP located in a window explaining the maximum variance in protein content in this group (3.97%). KMT5B is a histone methyltransferase that trimethylates ‘Lys-20’ of histone H4 (a tag for epigenetic transcriptional repression) and plays a role in myogenesis (Neguembor et al., 2013). Four SNPs in a gene that codes for SNW domain-containing protein 1 were identified. This protein positively regulates histone H3-K4 methylation (Brès et al., 2009). A single SNP was identified on ribosomal oxygenase 1 that functions as histone lysine demethylase, a ribosomal histidine hydroxylase, and contributes to MYC-induced transcriptional activation (Eilbracht et al., 2004; Suzuki et al., 2007; Ge et al., 2012). Two SNPs were identified in a gene coding for host cell factor 1 (HCF-1). In human, the cell-proliferation factor HCF-1 tethers Sin3 histone deacetylase and Set1/Ash2 histone H3-K4 methyltransferase (H3K4me) complexes that are involved in repression and activation of transcription, respectively (Wysocka et al., 2003). The list includes two other genes that harbored four SNPs on chromosome 3; transcription and mRNA export factor ENY2-2 and ubiquinone biosynthesis *O*-methyltransferase, mitochondrial.

Taken together, our results suggest that calcium homeostasis, more likely through RYR3, and transcriptional/chromatin regulators have major roles in regulating genetic variability in muscle protein content.

### Single Marker Association Analyses

**FIGURE 4:**
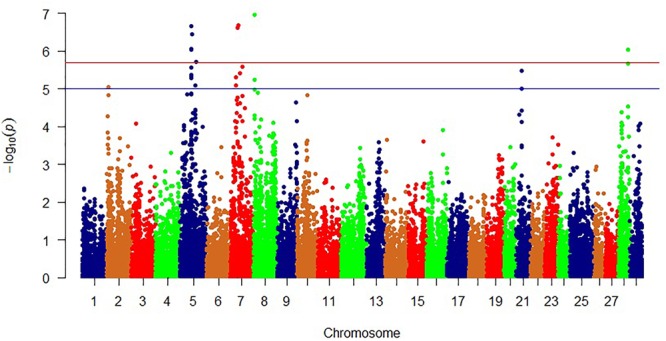
Manhattan plot showing single SNP markers associated with variations in shear force. Blue and red horizontal lines represent suggestive and significance threshold *p*-values of 1e-05 and 2.01e-06, respectively.

In addition to WssGBLUP and to identify single SNP marker association with phenotypic variation in shear force and protein content, we analyzed SNPs included in the SNP chip using general linear regression model available in PLINK which allows for multiple covariates (Purcell et al., 2007). In this study, PLINK identified 11 significant SNPs with potential impact on the shear force (Bonferroni-corrected *p* < 2.01E-06; Figure 4 and Table 5).

**Table 5:** SNP markers significantly associated with phenotypic variability in shear force using a single SNP marker analysis.

RefSeq	CHR	SNP position	Gene ID	Strand	Gene annotation	Region	*R*^2^	UNADJ	BONF
NC_035084.1	8	5559245	LOC110529177	–	Ryanodine receptor 3	CDS	0.034	1.08E-07	2.69E-03
NC_035083.1	7	27549740	LOC110527679	–	Cytochrome c oxidase subunit 6C-1	5′UTR	0.031	2.10E-07	5.22E-03
NC_035083.1	7	27591587	LOC100136192	–	14-3-3B1 protein	3′UTR	0.031	2.10E-07	5.22E-03
NC_035083.1	7	28432371	LOC110527692	+	Rho GTPase-activating protein 15	3′UTR	0.031	2.10E-07	5.22E-03
NC_035081.1	5	39867207	LOC110523898	+	Disabled homolog 2	3′UTR	0.026	2.24E-07	5.57E-03
NC_035083.1	7	24880637	pigu	–	Phosphatidylinositol glycan anchor biosynthesis class U	3′UTR	0.031	2.37E-07	5.89E-03
NC_035081.1	5	41247573	tcp4	–	Activated RNA polymerase II transcriptional coactivator p15	CDS	0.025	3.53E-07	8.79E-03
NC_035081.1	5	39901639	LOC110523901	+	Uncharacterized LOC110523901	lncRNA	0.011	8.72E-07	2.17E-02
NC_035104.1	28	34338597	LOC110509080	–	Annexin A13	CDS	0.020	9.39E-07	2.34E-02
NC_035081.1	5	39866812	LOC110523898	+	Disabled homolog 2	CDS	0.008	9.42E-07	2.34E-02
NC_035081.1	5	56278406	LOC110524193	–	Nicotinamide riboside kinase 2	3′UTR	0.030	1.96E-06	4.88E-02

*SNPs are sorted according to their *R*^*2*^ values.*

Most of the significant SNPs were located on chromosome 5 (*n* = 5) and chromosome 7 (*n* = 4). However, the most significant SNP explaining 3.4% of the phenotypic variability in shear force, was located on chromosome 8 in a gene coding for RYR3. This result was in agreement with the WssGBLUP 50 SNP-window analysis and suggests an essential role for RYR3 in regulating filet firmness in trout. Cytochrome c oxidase subunit 6C-1 (COX6C1), 14-3-3B1 protein, and rho GTPase-activating protein 15 (ARHGAP15) were ranked next to RYR3 in impacting phenotypic variability in shear force. COX6 was rapidly degraded under endoplasmic reticulum stress conditions induced by Ca^2+^ depletion (Hong et al., 2016) and upregulated in rainbow trout families of high shear force (Ali et al., 2018). 14-3-3B1 protein has been reported to be involved in apoptotic process (Rodrigues et al., 2017). Previous studies elucidated the involvement of 14-3-3 proteins in meat tenderness (Rodrigues et al., 2017). Overexpression of ARHGAP15 increases actin stress fibers and cell contraction (Seoh et al., 2003). ARHGAP15 SNP was in strong LD (*D*’ = 1), with two SNPs located in COX6C and 14-3-3B1 protein. In addition to 14-3-3B1 protein, a gene coding for disabled homolog 2 (DAB2) was also involved in apoptotic process (Prunier and Howe, 2005). Two SNP-harboring genes, phosphatidylinositol glycan anchor biosynthesis class U (PIGU) and annexin A13, were involved in lipid metabolism. PIGU has functions in lipid metabolism including membrane lipid biosynthesis. This gene exhibited differential expression in porcine muscles divergent for intramuscular fat, which correlates positively with meat tenderness (Hamill et al., 2013). Annexins are Ca^2+^-dependent phospholipid-binding proteins that have an important role in the cell cycle and apoptosis (Mirsaeidi et al., 2016). The list also includes a cell adhesion receptor, nicotinamide riboside kinase 2, that modulates myogenic differentiation (Li et al., 1999). Single-marker analysis did not identify SNPs in significant association with variation in protein content.

Altogether, results obtained from the single SNP analyses provided additional evidence of RYR3 role in regulating phenotypic variability in filet firmness. Also, single-marker analysis highlighted a role for a few more genes in filet firmness. However, estimating the effect of each SNP individually does not allow the detection of small effects of multiple joint SNPs. This may explain the inconsistency in the significant peaks between the single-marker analysis and the WssGBLUP approach. Several studies indicated that the SNP-joint analysis is more successful than the single-SNP analysis in GWA studies of complex traits (Fridley and Biernacka, 2011; Lu et al., 2015). Therefore, WssGBLUP approach is assumed to be more effective in dissecting the genetic architecture of the studied traits and providing putative markers that can be used for selection purposes.

## Conclusion

The current GWA analyses identified novel genomic loci with a role in regulating muscle firmness and protein content. These genomic loci code for proteins involved in calcium homeostasis, transcriptional and chromatin regulators, cell adhesion, protein synthesis/degradation, and apoptotic processes. The top windows affecting the additive genetic variance in protein content and shear force appeared on chromosome 4 and 13, respectively. RYR3 was the major gene harboring the largest number of SNPs located within windows affecting the additive genetic variance in shear force and protein content. Abnormal calcium homeostasis in muscle cells accelerates postmortem protein degradation, and meat softness (Barbut et al., 2008). The current study revealed that WssGBLUP, using 50 adjacent SNP windows, provided putative markers that could be used to estimate breeding values for firmness and protein content.

## Data Availability

All datasets generated for this study are included in the manuscript and/or the Supplementary Files. The genotypes (ped and .map files) and phenotypes are available in Supplementary Data Sheet S1. A list of all SNPs affecting the additive genetic variances are provided in Supplementary Tables S7, S8.

## Author Contributions

MS, TL, and BK conceived and designed the experiments. RA-T, MS, TL, and BK performed the experiments. RA-T, AA, DL, BK, and MS analyzed the data. AA and MS wrote the manuscript. All authors reviewed and approved the publication.

## Conflict of Interest Statement

The authors declare that the research was conducted in the absence of any commercial or financial relationships that could be construed as a potential conflict of interest.

## Supplementary Material

TABLE S1 List of all SNPs affecting >2% of the additive genetic variance in shear force.

TABLE S2 Created/deleted miRNA targets as a consequence of SNPs affecting genetic variance in shear force.

TABLE S3 David functional annotation for genes harboring SNPs affecting the additive genetic variance in shear force.

TABLE S4 List of all SNPs affecting >2% of the additive genetic variance in protein content.

TABLE S5 Created/deleted miRNA targets as a consequence of SNPs affecting genetic variance in protein content.

TABLE S6 David functional annotation for genes harboring SNPs affecting the additive genetic variance in shear force.

TABLE S7 List of all SNPs affecting the additive genetic variance in shear force.

TABLE S8 List of all SNPs affecting the additive genetic variance in protein content.

DATA SHEET S1 The genotypes (ped and map files) and phenotypes files used in GWA analysis.
